# Prosecution of Malpractice

**Published:** 1855-12

**Authors:** A. Moore

**Affiliations:** Littleton, N. H.


*Prosecution for Malpractice, in a Case of Imperfect Recovery from a Dislocation of the Elbow.*

*Farr* vs. *Moore*, Grafton County, New Hampshire. The case was tried at the April Term, 1853, before Mr. Justice Woods. H. & G. A. Bringham for plaintiff, C. W. Rand and H. A. Bellows for defendant.

On the 17th day of September, 1850, the plaintiff, a boy nine years old, was running and fell, dislocating the forearm backwards.

Dr. Adams Moore, of Littleton, who lived at the village, nine miles from the patient's home, was called, and arrived about three hours after the injury happened.

He found the joint much swollen and very painful. The arm was easily drawn into place with a slight chuck. On flexing the arm the boy complained very much.

The case was regarded and treated as a simple backward dislocation, with more than ordinary swelling and pain. The swelling and extreme sensitiveness of the boy, and his absolute refusal to inhale chloroform, prevented a minute examination.

The boy was born of a scrofulous mother, whose lungs and general health had been impaired before his birth, and who died of cancer of the breast. He had a cleft palate; bore the aspect of a strongly marked scrofulous constitution; and did not manifest the ordinary degree of mental development of boys of his age.

He was lying on a bed when the dislocation was reduced, and was left there with the arm in a semiflexed position, resting on a pillow, covered with a napkin saturated with cold water. The use of bandages, splint, and sling, was considered improper. The application of cold water, with entire rest, was directed. The father was also told not to move the joint for eight or ten days, and then to begin motion without any efforts of the boy.

No request was made to the surgeon to visit the boy again, the surgeon saying it did not appear necessary The father undertook the care of it himself.

The following night the limb continued to swell, and was quite painful, although the boy took morphine.

In the forenoon of the next day, the father applied to Dr. Burns, of

Littleton, the senior physician of that region, who visited the boy with Dr. Moore.

After hearing a history of the case from the friends and Dr. Moore, and examining it as far as he thought proper, he gave it as his opinion that there was no appearance of any existing displacement of the bones; and advised a continuance of the treatment first prescribed, until the inflammation and swelling should subside; and if then all did not appear right, to bring the boy down to the village for further examination.

No more was heard from the boy until the seventeenth day after the injury. At that time the father took the boy to Dr. Burns for examination. Dr. Burns looked at it, told the father to ask Dr. Moore to come to his office, at the same time saying that the arm was not right, which observation was reported to Dr. Moore at the time.

He went to Dr. Burns' office, and found the arm capable of only partial flexion and extension. The covering of the joint was thickened and indurated all around. The prone and supine motions of the fore-arm were perfect; and no displacement of any bone could be discovered by Dr. Moore. As Dr. Burns had expressed an opinion conveying the idea that the dislocation had not been perfectly reduced, he was specially requested by Dr. Moore to point out wherein there was any bone in a wrong position. He examined it thoroughly, and admitted that he could not discover any displacement. Friction, flexion, and extension of the arm, as forcibly as the boy could bear, were advised.

About three weeks after, the father again brought the boy to Dr. Burns, who sent for Dr. Moore.

No material change had taken place, except the abatement of soreness. The motion of the joint, and the thickening of the integuments, were much as before. The sorest part was in the bend of the arm over the coronoid process, and there the thickening of the parts was the greatest. There was clearly some deep injury. It was proposed to administer chloroform to the boy and make a more thorough examination, which was done next day at the home of the boy. Extension, flexion, and a thorough manipulation for a considerable time were made, but no displacement could be discovered. An internal lateral displacement of the ulna had been suspected by Dr Moore, who at this time became satisfied that it did not exist. A fracture of the coronoid process was supposed be possible, but the rigid state of the parts prevented any satisfactory conclusion.—Nothing new was advised, but to continue the treatment last recommended. The father had become dissatisfied, and was talking about his right to damages. About six months after the injury, the boy was presented to Dr. Dixi Crosby, Professor of Surgery in Dartmouth College, who was then at a railroad depot, waiting the arrival of the train, and had not time for a deliberate examination. He made an examination, and suggested that the ulna might be displaced inwardly; and soon found from the father that he was after an opinion for the purpose of sustaining a suit. He told him decidedly that he had no reasonable ground for such a claim.

On reaching home, Dr. Crosby wrote to Dr. Moore, stating what had transpired, and advising a careful examination in relation to an internal displacement of the ulna, which he was inclined to think might be found. As such an examination had already been made, while the boy was insensible to pain, the examination was not repeated.

About this time two suits were instituted against Dr. Moore. One in the name of the boy, by his father as guardian, on account of the loss of the use of his arm ; and one by the father, for expenses and loss of the service of the boy ; both claiming heavy damages.

Dr. Burns was not included. The opinion he had expressed might be of service in evidence.

About a year after the accident, Dr. Burns was called by the plaintiff to depose as to his knowledge of the matter and the present state of the arm.

He stated then that "the bones don't appear to be in the proper place —it" (the arm) "is very much enlarged about the joint"—"the appearance is that the joint is not in its proper place."

On cross-examination, he was requested to point out any bone connected with the joint, which was not in its natural position. To this he replied that he "did not know as he could."

At this time there was a slight crackling in the joint when moved ; and the arm could be bent to nearly a right angle with the upper bone.

Dr. Burns afterwards, from a more careful examination of the subject in reference to there having been a fracture of the coronoid process, entirely changed his opinion of there being any unreduced dislocation.

About two years after the accident, Dr. C. M. Tuttle, of Littleton, went and examined the joint, and compared the appearances with those of the case of Sir Astley Cooper, as described in his book, and came to the conclusion that it was an injury of that kind, and that the recovery in this case was as good as that is represented to have been.

About the same time, Dr. E. R. Peaslee, Professor of Anatomy, &c., in Dartmouth College, was called upon by the plaintiffs to examine the arm, and testify as to its condition.

After a careful examination of the boy alone, he stated that the following was the condition of the limb :

"I find some difficulty at the elbow joint. In the first place there is a defect in extension ; secondly, there is a defect in flexion (the arm not being bent beyond about a right angle ;) thirdly, there is some deformity at the joint ; fourthly, the ulna is somewhat displaced backwards ; fifthly, the radius is also slightly displaced ; sixthly, the head of the radius is enlarged." The displacement of the radius is "backwards and downwards, being slight," and that the head of the radius was "very near on its natural articulating surface, but not precisely ;" "that he could discover no lateral displacement of the ulna." He said that a partial dislocation of the radius backward was not an accident recognized by surgeons as he was aware, unless complicated with a fracture of the coronoid process ; and that in this case it was "altogether probable that the coronoid process had been broken ;" and that the boy bore the aspect of a highly scrofulous constitution.

Soon after this, the boy was taken by the father, accompanied by his counsel, for an *ex parte* opinion to Dr. J. P. Bancroft, of St. Johnsbury, Vermont. Dr. Bancroft says he made the examination and declined giving an opinion until he had time to refer to his books, which he did, and came to the conclusion that it was a case of fracture of the coronoid process ; and that he never had had experience in such a case. He also said that a scrofulous diathesis was a serious impediment to complete recovery in injuries of joints.

The physicians already named gave depositions out of court to be used on the trial.

Also, Dr. E. Dyman, of Lancaster, who had been in practice fifty years, was applied to by the plaintiffs. He had examined the arm a few months after the injury, and also examined it when he gave his deposition about a year after the accident. He, without hesitation, called it "a case of bad surgery;" said the ulna was in place, but that "the radius was thrown off, as he conceived it."

The trial in court was a long one; nearly a week was spent in presenting the testimony to the jury. Preparation was made to appeal to the sympathies of jurors. The boy-plaintiff appeared with his counsel accompanied by his young married sister with a nursing child, unattended by their father.

The plaintiff in his opening took the position that the defendant had made a mistake in supposing at first that the case was one of simple dislocation; and also that in treating it as such, he had not proceeded right in not applying bandages, splints, and a sling, and securing the arm bent to a right angle with the humerus.

Another point was that he had made another mistake in altering his mind and thinking that it was complicated with a fracture of the coronoid process, and undertook to prove that the original accident was a fracture of one of the condyles. Another point was that the defendant had neglected the boy because his father was poor and did not pay his doctor's bills.

To sustain the first two positions, he asked leave to introduce the book of Sir Astley Cooper on "Fractures and Dislocations." The defendant waived all legal objection to the use of the book; and the court allowed either party to read from the book such parts as he chose, thus making Sir Astley Cooper a witness in the case for the plaintiff.

The counsel for the plaintiff then read Sir Astley's directions for treating a simple backward dislocation of the forearm; after reduction, "to bandage the arm in the bent position, the limb to be supported in a sling; the forearm to be bent rather less than a right angle with the upper arm; a splint placed in the sling for the better support of the limb." Nothing of this sort was done in this case.

The plate of Sir Astley, representing such a dressing, was exhibited from the book to the jury.

On the second position, that the original injury was a fracture, he read the remarks that oblique fractures of the condyles are often mistaken for dislocations, and happen more frequently in children than in persons of advanced age; and as an evidence of a fracture which had never united, he exhiflited the crackling sound in the joint as crepitus of bones; and that such an injury could not be properly treated without bandages and splints.

On the third point, the saying of the defendant at the time of his first visit that he did not think it necessary to come again, was evidence of indifference and neglect.

The first of these allegations, "that the case was taken at first to be one of simple backward dislocation," was not denied by the defendant, but he contended that he had good reason to suppose it was so; a fracture of the coronoid process being an exceedingly rare accident, that Sir Astley Cooper, in all his immense practice, had noticed it only once, and that in a patient sent him from the country; and in this case it was impossible to discover such an injury at the time, without a handling too severe to be justified.

As to not using bandages, splints, a sling, and confining the arm in a bent position, he said they were not essential to a good recovery in a simple backward dislocation of the forearm, and were used only as contributing to the comfort of the patient in carrying the limb; and further, that in all cases where surgeons direct their use, it is understood that there is no great degree of inflammation—that in highly inflamed limbs they would be injurious. The 261st case from the plaintiff's book was read, where Sir Astley Cooper, after reducing the dislocation, laid the arm on a pillow, with a poultice—inflammation supervened—in four days the integuments from wrist to shoulder became gangrenous, and the patient died the same evening.

The plaintiff himself proved that the boy's arm swelled above and below the elbow, and "shined like a glass bottle."

The defendant contended that after reduction, followed by severe inflammation, as in this case, the inflammation became the paramount disease; and that Sir Astley's book on "Fractures and Dislocations" did not treat specially of "Inflammation."

Dr. Hibbard, of Lisbon, explained to the jury the proper treatment in such cases, and the great danger of compressing inflamed parts by splints and bandages.

In answer to the noise in the joint being evidence of a fracture ununited, Dr. Morgan, of Haverhill, testified that it was common in cases of injured joints, and very different from crepitus. He also read to the jury out of the plaintiff's book that "some time after the accident" (dislocation) "it frequently happens that a sensation of crepitus is produced by effusion of adhesive matter into the joint and surrounding bursæ; the synovia in which becomes inspissated and crackles under motion. But every practitioner ought to be able to distinguish this crackling from the grating crepitus of fracture." Also that "the degree of inflammation which succeeds to these accidents is generally slight, but in some cases it is very considerable, and produces great tumefaction, which added to that resulting from extravasation of blood, frequently renders the detection of the injury exceedingly difficult."

Dr. Crosby was called before the court and requested to examine the arm, to enable him to testify. He declined making any examination for the purpose of obtaining knowledge to enable him to testify. but was ready to answer all questions so far as his present knowledge should enable him; unless the court was of opinion that the law required him to make an examination for that purpose. The judge ruled that physicians are required by law to do so, as an expert would be required to look upon a bank note and say whether it is genuine.

Dr. Crosby then made the examination, and statad that the limb bent much better than it did when he first examined it about two years before. He thought the difficulty might be owing to the rigidity of the ligaments; that he could not satisfy himself that there was any displacement of the bones of the forearm; that there was no evidence of any fracture of a condyle; could not tell whether there had been a fracture of the coronoid process—regarded it to be very difficult to detect a fracture of that process in a case of dislocation; could not tell what caused the stiffness of the joint; had thought on his first examination, and thought now, that the articulating end of the humerus, the trochlea, might have been broken, and thought the deformity might be accounted for in this way better than any other. He had never found any fracture of the coronoid process, still it might exist here. He thought the reduction of the dislocation had been properly made.

The physicians all testified that rest and cold evaporating lotions were the main elements in treatment of dislocations after reduction of the fore-arm. They also testified that fractures of the coronoid process were very rare, no one having regognized that accident in his practice; and further, that recovery after it would probably be always imperfect, it being generally believed that union between the broken fragments would be by ligaments, if at all.

Dr. P. Spalding, of Haverhill, testified that the arm above the elbow was three-fourths of an inch enlarged. The head of the radius appeared a little raised; that when the arm was extended, the olecranon was carried from the external condyle of the humerous about one-half an inch, and yet the distance from the olecranon to the extreme point of the internal condyle was the same in both arms; that the condyles of the humerus by admeasurement were precisely the same as to their extreme points; and that the arm when extended seemed to permit a straight elevation of the olecranon from the fossa of the humerus; that pronation and supination of the radius were perfect; that when the arm was nearly extended the tendons of the biceps and brachialis muscles were put to the utmost stretch; and that when the arm was flexed to a right angle, the ulna seemed to come in contact with a solid substance in the hollow of the arm, so as to prevent entirely any further flexion.

He gave it as his opinion that there had been a dislocation of the elbow backwards, together with fracture of the coronoid process of the ulna; and suggested that the broken point had adhered to the trochlea, and had not united with the ulna.

As to negligence on the part of the surgeon, the plaintiff, in his zeal to make the time as short as possible between that of the accident and the arrival of the doctor, made it appear that the surgeon rode from his home to that of the patient, nine miles, over a rough uphill road, in one hour (which was really straining the truth, but it showed no lack of promptness on his part.) The defendant also offered to show the declaration of the farther that he believed Dr. Moore did the best he could, and that he never charged him otherwise; that all he blamed him for, was that he would not acknowledge the arm wrong when Dr, Burns said it was, and he must have known it was. To the admission of this testimony the plaintiff's counsel objected, the boy being the party to the suit, and not the father. The judge was not clear that it was proper, and the defendant's counsel thought it best not to put it in, as it might operate to set aside the verdict, in the event he obtained it.

The judge in his charge to the jury observed that the having a bad arm is not a light matter, and that the reputation of this defendant is also not a light matter.

The plaintiff alleges that he procured the attendance of Dr. Moore, that he did not set the limb properly, that he did not bandage the arm, or treat it properly afterwards.

The defendant alleges that there was a dislocation backwards, which he supposed to be simple, but finally found to be complicated with a fracture of the coronoid process, or of the trochlea of the humerus.

That at his first visit appearances did not require him to visit it again, that splints and bandages were not proper—that the inflammation was such as would have made them injurious.

That fracture of the coronoid process, or of the trochlea, is never followed by a perfect recovery. That there were depositions within and around the joint, caused by the inflammation; and that the stiffness consequent upon these deposits may, and very probably is an important part of the difficulty.

There is no doubt that the boy is incurably lame, but from what cause does it arise? If the surgeon exercised reasonable care and skill in the case, he is not liable. He is not obliged to guarantee a perfect cure.

It is charged that he was negligent. If he supposed it to be a simple dislocation, and that it was reduced, was there any necessity of his going again? Was it proper that he should direct to bring the boy down to the village, when the inflammation had subsided? Does the fact that he went there in one hour show that he was prompt and attentive? and the fact that he took Dr. Burns, an old and experienced physician, with him, show that he was careful, and wanted to treat it carefully?

You are to decide whether the permanent lameness of the plaintiff is caused by the fractures or deposits, or by the want of skill and care on the part of the surgeon. The jury, after deliberating a long time, reported that they could not agree, and were discharged. It was rumored abroad afterwards that they stood three for plaintiff and nine for defendant. It was further said that two of the minority would agree with the nine for the defendant, if the *one* would. Before the next term of court, the plaintiffs became nonsuit by agreement.

Some of his medical brethren expressed a wish at the time of trial to know the defendant's own opinion in regard to the real nature of the injury, as he carefully avoided expressing any that might at all influence theirs, while the matter was undergoing a legal investigation. His opinion is based on the four following facts, three of which were unknown to them:

1. The bones of the forearm were drawn into place with more ease than was expected—the chuck, as they slipped into place, though distinct, was less so than usual, but did not raise the idea of a fracture at the time.

2. The bending of the arm caused a considerable increase of pain; the limb being much swollen and the boy very sensitive, this was not regarded as evidence of a fracture.

3. There was for a considerable time not only a very decided thickening of the integuments directly over the coronoid process, but the tenderness at that point was greatest.

4. When the arm is flexed, the ulna and radius seem to be in place; when extended, the point of the ulna strikes a little back and to one side of the posterior fossa, showing a slight backward slipping, for the want of a full support of a perfect process in front, and drawing the radius with it, the circular ligament probably being not broken.

That the broken point of the coronoid process has adhered to the front of the trochlea, or some solid growth thrown out there against which the remainder of the process strikes on flexion, appears to him very probable.

When driven to a careful investigation of the subject of fractures of the coronoid process, the defendant was led to notice the two following statements in standard surgical works, which are calculated to mislead, and may be seized upon by plaintiffs' counsel to operate on the minds of jurors:

Mr. Fergusson, in his *Operative Surgery*, says of backward dislocation of both bones of the forearm: "In such a case, the coronoid proces will *probably* be broken." This was urged in the trial by plaintiff's counsel as contradicting the medical testimony, and charging a conspiracy on the part of the doctors to defeat the ends of justice, in saying that the accident was very *rare*.

One other case came to the notice of the defendant, but not to the plaintiff's counsel:

Dr. Dorsey, in his *Surgery*, says that Dr. Physick once saw a case of fracture of the coronoid process; that the forearm was kept fixed at a right angle with the humerus, and the tendency of the brachialis muscle to draw up the superior fragment was counteracted in some measure by pressure of the roller above the elbow, and "that a *perfect cure* was *readily obtained*."

In the first, perhaps the word "possibly" has given place to "probably."

In the latter, it may well be doubted whether the case is fairly represented by Dr. Dorsey, it not having come within his own observation.

A. Moore.

[*American Medical Monthly.*

Littleton, N. H.

